# Plasma Vitamin B12 Levels, High-Dose Vitamin B12 Treatment, and Risk of Dementia

**DOI:** 10.3233/JAD-201096

**Published:** 2021-02-16

**Authors:** Johan Frederik Håkonsen Arendt, Erzsébet Horváth-Puhó, Henrik Toft Sørensen, Ebba Nexø, Lars Pedersen, Anne Gulbech Ording, Victor W. Henderson

**Affiliations:** a Department of Clinical Epidemiology, Aarhus University Hospital, Aarhus, Denmark; b Department of Clinical Biochemistry, Aarhus University Hospital, Aarhus, Denmark; c Department of Epidemiology & Population Health, Stanford University, Stanford, CA, USA; d Department of Neurology & Neurological Sciences, Stanford University, Stanford, CA, USA

**Keywords:** Alzheimer’s disease, cobalamin, cohort studies, clinical nutrition, registries

## Abstract

**Background:**

It is controversial whether B12 deficiency causes dementia or B12 treatment can prevent dementia.

**Objective:**

To assess associations between low plasma (P-)B12 levels, B12 treatment, and risk of Alzheimer’s disease (AD; primary outcome) and all-cause or vascular dementia (secondary outcomes).

**Methods:**

We conducted a population-based cohort study using Danish registry data to assess associations between low P-B12 levels, high-dose injection or oral B12 treatment, and risk of dementia (study period 2000–2013). The primary P-B12 cohort included patients with a first-time P-B12 measurement whose subsequent B12 treatment was recorded. The secondary B12 treatment cohort included patients with a first-time B12 prescription and P-B12 measurement within one year before this prescription. For both cohorts, patients with low P-B12 levels (<200 pmol/L) were propensity score-matched 1:1 with patients with normal levels (200–600 pmol/L). We used multivariable Cox regression to compute 0–15-year hazard ratios for dementia.

**Results:**

For low P-B12 and normal P-B12 level groups, we included 53,089 patients in the primary P-B12 cohort and 13,656 patients in the secondary B12 treatment cohort. In the P-B12 cohort, hazard ratios for AD centered around one, regardless of follow-up period or treatment during follow-up. In the B12 treatment cohort, risk of AD was unaffected by low pre-treatment P-B12 levels, follow-up period and type of B12 treatment. Findings were similar for all-cause and vascular dementia.

**Conclusion:**

We found no associatio1n between low P-B12 levels and dementia. Associations were unaffected by B12 treatment. Results do not support routine screening for B12 deficiency in patients with suspected dementia.

## INTRODUCTION

Vitamin B12 (B12) is essential in DNA synthesis, and in both fatty acid and amino acid metabolism. It is important in the maturation of hematopoietic cells and in the normal functioning of the nervous system via its role in myelin synthesis [1].

Low plasma B12 (P-B12) is associated with various neurological disorders including dementia, but two systematic reviews concluded that findings were inconsistent [2, 3]. Some individual studies showed that biomarkers of B12 deficiency—low P-B12, high P-homocysteine, and high P-methylmalonic acid (P-MMA)—were associated with poor cognitive scores and risk of dementia, including Alzheimer’s disease. However, both reviews questioned whether B12 is important for cognitive decline and dementia, mainly because study populations were small and of short follow-up durations.

Although, high-dose B vitamins that include B12 might reduce cerebral atrophy [4, 5], meta-analyses of trials report that oral B vitamin treatment does not prevent cognitive decline or Alzheimer’s disease [6–9]. These meta-analyses, however, were criticized for including patients at low risk for B12 deficiency [10]. Moreover, no study has assessed whether oral and parenteral B12 treatment have different effects on Alzheimer’s disease risk. The global burden of dementia is increasing [11], supporting the importance of valid preventive measures, including nutritional supplements and the need for tests to screen for so-called treatable dementias.

To this end, we assessed the risk of Alzheimer’s disease and other dementia types in a population-based cohort of Danish patients with various levels of P-B12 and P-MMA and evaluated the potential protective effect of treatment with high-dose B12 on dementia risk, including the specific effects of three different B12 formulations.

## METHODS

### Data sources

We conducted a population-based, propensity score-matched cohort study in the Northern and Central administrative regions of Denmark using Danish health registries. These two regions cover a population of about 1.8 million persons, approximately a third of the Danish population. The tax-supported Danish health care system provides free access to general practitioners, specialists and hospital care. The Danish Civil Registration System [12] assigns a unique 10-digit identifier (CPR number) to all Danish residents at birth or upon immigration, and this identifier was used for linkage of individual patient data across the registries. Data from the following sources were used (see Supplementary Table 1 for specific codes).

The LABKA database [13] contains biomarker results from clinical laboratories at all hospitals in Northern and Central Denmark, starting in 1998. Biomarker results are available for inpatients, hospital outpatients, and for patients consulting both specialist and general practitioners in the study area. The results are coded according to the Nomenclature, Properties and Units system, with each record containing the code (or modified Danish code), the date and time of measurement, the patient’s CPR number, and the test results (or an indicator for a missing result).

The Aarhus University Prescription Database [14] (AUPD) contains data on all reimbursed prescriptions in Northern and Central Denmark since 1998. Most prescriptions are eligible for either full or partial reimbursement to individual patients and are therefore recorded in the database. The record for each reimbursed prescription contains the patient’s CPR number, the date of dispensing, identification of the prescribing physician hospital or department, the name, pack size, and manufacturer of the drug, and the ATC code used to identify the drug.

The Danish National Prescription Registry [15] holds data on redeemed prescriptions in the entire Denmark since 1995, including non-reimbursed prescriptions. Each record holds similar information as in the AUPD. Approximately 15% of B12 prescriptions in Denmark are redeemed without reimbursement. We only had limited access to the Danish National Prescription Registry, so this registry was only available for sensitivity analyses.

The Danish National Patient Registry [16] (DNPR) records hospital admissions in Denmark since 1977 and emergency department and outpatient clinic visits since 1995. The treating physician submits a primary diagnosis as the primary reason for hospital contact and up to 20 secondary diagnoses, coded according to the International Classification of Diseases, Tenth Revision (ICD-10) (ICD-8 was used through 1993).

The Danish Psychiatric Central Research Register [17] (DPCRR) includes data on admissions, diagnoses, and treatments at Danish psychiatric hospitals since 1970. It was merged with the DNPR in 1995, and diagnoses are also recorded in ICD codes.

The Danish Register on Personal Income [18] holds data on personal income from 1970 and onwards. Personal income is recorded in detail and also includes sickness and unemployment benefits and early and state pension.

Data on education from the Danish Education Register [19] is collected at the individual level and considered complete and valid.

Data on employment status at the individual levels was collected from the Danish Register on Labor Market Affiliation [20]. This register is also considered complete and of high validity.

### Study cohorts

#### Primary P-B12 cohort

We selected a primary P-B12 cohort with a first-time P-B12 measurement in LABKA from January 1, 2000 to December 31, 2013, with the date of P-B12 measurement serving as the index date.

We defined low P-B12 as <200 pmol/L (the lower reference limit for the population) and normal P-B12 as 200–600 pmol/L. We used propensity scores to match patients with low P-B12 values in a 1:1 ratio to patients with normal P-B12 values (see *Propensity Score Matching).*

#### Secondary B12 treatment cohort

A secondary B12 treatment cohort was sampled using the date of the first B12 drug prescription recorded in the AUPD as the index date in the period from January 1, 2000 to December 31, 2013. Patients were included if they had a P-B12 measurement within 1 year prior to the index date. We used data for the three types of B12 drugs available to Danish patients: oral cyano-B12 1 mg/day; injectable cyano-B12 1 mg in oil suspension, injected every third month; and injectable hydroxo-B12 1 mg in aqueous solution, injected every second to third month. All three treatments are considered high-dose treatments [21], since the recommended daily intake of B12 is only 2–4*μ*g [1]. We were unable to assess whether dosage or injection intervals followed the medical recommendations. We used propensity scores to match patients with low pre-treatment P-B12 values in a 1:1 ratio to patients with normal pre-treatment P-B12 values.

### Exclusion criteria

We used the following exclusion criteria for both cohorts: 1) Age <40 years; 2) P-B12 levels >600 pmol/L, since high values may reflect severe disease, including cancer or liver disease [22, 23]; 3) Diagnosis of dementia of any cause or of mild cognitive impairment or amnestic syndrome, which may indicate impending dementia, recorded in DNPR or DPCRR at any time prior to the index date for each of the two cohorts; 4) Redemption of any prescription for B12 drugs recorded in the AUPD up to two years before the date of the P-B12 measurement.

### Outcome

The main outcome was Alzheimer’s disease and the secondary outcomes were all-cause dementia and vascular dementia recorded in the DNPR. All-cause dementia was defined as a code for either Alzheimer’s disease, vascular dementia, or another dementia type [24].

All members of the patient cohorts were followed until the date of an incident dementia diagnosis, emigration, death, or December 31, 2015, whichever came first.

### Study variables

#### Propensity score matching

The propensity score included diseases that are potential risk factors for dementia and diseases other than dementia that are associated with B12 deficiency. Data on diseases were collected as diagnosis codes recorded in the DNPR or DPCRR and prescriptions contained in the AUPD for drugs used to treat these diseases (see Supplementary Table 1 for specific ICD and ATC codes).

We performed the propensity score matching by generating a multivariable logistic regression model that predicted low P-B12 levels conditional on the covariates included in the multivariable model. The following variables were included in the model: gender, year of P-B12 measurement, age, and the following diseases: cardiovascular diseases, gastrointestinal diseases, neurological infections, Parkinson’s disease, epilepsy, and psychiatric diseases. We then computed the probability of low P-B12 levels (= the propensity score) for all patients and visually illustrated the propensity score distribution among low and normal P-B12 levels. Using a nearest neighbor matching, we matched each participant with low P-B12 levels with the closest possible normal P-B12 levels group participant. The matching was performed without replacement, within a maximum matching range (caliper width) in propensity score of 0.2 times the standard deviation of the propensity score.

#### Covariates

The secondary B12 treatment cohort was further divided into three treatment sub-cohorts and propensity score matching was repeated according to the three types of B12 drugs. The analyses included the covariates describe below:

While P-MMA is a more sensitive marker for B12 deficiency than P-B12, it is less widely used than P-B12 due to laboratory costs [1]. In Northern and Central Denmark, a laboratory algorithm is used that automatically measures P-MMA if a P-B12 value is between 125–250 pmol/L [21]. Therefore, we included the P-MMA measurement closest to and within one year before the index date for the two cohorts from the LABKA database. Kidney disease may elevate P-MMA levels in the absence of B12 deficiency [1], so we also included the P-creatinine measurement closest to and within one year of the P-MMA measurement to exclude patients with kidney diseases.

Using the Danish Register on Personal Income, we categorized personal income in quartiles in the same year as the index dates for the two cohorts. The highest achieved education was obtained from the Danish Education Register and categorized as primary school, youth education, high school or similar education, and higher education (bachelor’s or higher degree). Employment status from the Danish Register on Labor Market Affiliation during the same year as the index date was categorized as employed, unemployed, early retirement, and state pensioner. Patients with missing socioeconomic data were excluded.

The Danish National Prescription Registry was used to capture data on the approximately 15% of B12 prescriptions that are redeemed without reimbursement. Due to data restrictions, we were unable to include these data in the phase of propensity score matching or as exclusion criteria in the initial creation of the two study cohorts. Hence, we did not use data on non-reimbursed B12 prescriptions to identify the secondary B12 treatment cohort, but only to capture B12 treatment during follow-up and for exclusion after propensity score matching in a sensitivity analysis in the primary P-B12 cohort.

### Statistical analyses

#### Main analysis

We computed incidence rates (IRs) per 1000 person-years of follow-up with 95% CIs according to the two groups defined by low or normal P-B12 levels.

We used a stratified Cox proportional hazards regression model to compute adjusted hazard ratios (HRs) with corresponding 95% CIs, treating patients with normal P-B12 values as reference. We adjusted for age, sex, income level, highest achieved education, employment status, and calendar year of the index date for the two cohorts.

Estimates were computed separately for the primary P-B12 cohort and the secondary B12 treatment cohort and stratified according to primary and secondary outcomes and years of follow-up (overall, 0–<2, 2–<7, and 7–15).

#### Subanalyses

First, we defined B12 treatment during follow-up in the primary P-B12 cohort as at least one B12 drug prescription in the AUPD after the index date to assess if B12 treatment during follow-up affected the association between low P-B12 and dementia outcomes. We computed IRs and HRs for both treated and untreated patients during follow-up, as described above.

Second, to assess any drug-specific effects, we repeated the analyses in the secondary B12 treatment cohort for each of the three B12 drug treatments.

Third, we excluded patients with kidney disease from the analysis of P-MMA by using the P-creatinine level to compute estimated glomerular filtration rates based on the modified Modification of Diet in Renal Disease Study equation [25]. Patients with estimated glomerular filtration rates <60 ml/min/1.73 m^2^ were excluded. Patients were categorized according to P-MMA levels in three categories: not B12 deficient: ≤0.28*μ*mol/L; possibly B12 deficient: 0.29–0.45*μ*mol/L; and B12 deficient: ≥0.46*μ*mol/L. We computed IRs with 95% CIs for Alzheimer’s disease according to P-MMA categories in both the two cohorts. Using patients with P-MMA values ≤0.28*μ*mol/L as the reference, we performed stratified Cox proportional hazards regression, adjusting for the same covariates as in the main analysis plus the variables used to define the diseases included in the propensity score. Both IRs and HRs were disaggregated according to years of follow-up.

#### Sensitivity analyses

20,036 patients were excluded from the secondary B12 treatment cohort due to incomplete propensity score matching. To assess the potential bias created by this exclusion, we analyzed the secondary cohort before propensity-score matching, including the drug-specific subanalysis. We used Cox regression analyses with additional adjustment for the same diseases that were included in the propensity score.

We added data on B12 drug prescriptions from the Danish National Prescription Registry to capture data on non-reimbursed B12 prescriptions for exclusion and to assess B12 treatment during follow-up in the primary P-B12 cohort.

All statistical analyses were performed using SAS version 9.4 (SAS Institute Inc., Cary, NC, USA). The study was approved by the Danish Data Protection Agency (record number: 2014-54-0922). Ethics approval is not needed for using registry data for research in Denmark [26].

## RESULTS

### Basic characteristics

The primary P-B12 cohort included 53,089 patients with a median age of 63 years in each of the two P-B12 level groups. Median follow-up time was 5.1 years (interquartile range: 2.8–7.6 years) for patients with normal P-B12 values and 5.0 years (interquartile range: 2.7–7.5 years) for patients with low P-B12 values. In total, 6.8% of patients with normal P-B12 levels and 48.6% of patients with low P-B12 levels received a B12 drug prescription during follow-up.

The secondary B12 treatment cohort consisted of 13,656 patients in each of the two P-B12 level groups. The median age was 65 years, and median years of follow-up were 4.5 years (interquartile range: 2.5–6.8 years) among patients with normal P-B12 values and 4.8 years (interquartile range: 2.6–7.2 years) among patients with low P-B12. For further characteristics of the two cohorts, see Table 1. For characteristics of the not-propensity score-matched secondary cohort, see Supplementary Table 2.

**Table 1:** Basic characteristics of the primary P-B12 cohort and the secondary B12 treatment cohort, Denmark, 2000–2013

	Primary P-B12 cohort P-B12 levels (pmol/L)		Secondary B12 treatment cohort P-B12 levels (pmol/L)	
	200–600	<200	200–600	<200
**Total**	53,089 (100.0)	53,089 (100.0)	13,656 (100.0)	13,656 (100.0)
**Sex, female**	29,033 (54.7)	28,807 (54.3)	8,292 (60.7)	8,365 (61.3)
**Age^a^**
40–60 y	22,695 (42.7)	22,791 (42.9)	5,395 (39.5)	5,208 (38.1)
61–80 y	24,262 (45.7)	23,906 (45.0)	6,167 (45.2)	6,548 (47.9)
≥81 y	6,132 (11.6)	6,392 (12.0)	2,094 (15.3)	1,900 (13.9)
**Year of P-B12 measurement/B12 treatment**
2000–2004	10,068 (19.0)	10,084 (19.0)	2,600 (19.0)	2,490 (18.2)
2005–2009	23,156 (43.6)	23,054 (43.4)	5,736 (42.0)	5,846 (42.8)
2010–2013	19,865 (37.4)	19,951 (37.6)	5,320 (39.0)	5,320 (39.0)
**Died during follow-up**
No	43,536 (82.0)	43,038 (81.1)	10,272 (75.2)	10,813 (79.2)
Yes	9,553 (18.0)	10,051 (18.9)	3,384 (24.8)	2,843 (20.8)
**Previous diagnoses**
Cardiovascular diseases	32,611 (61.4)	32,440 (61.1)	10,529 (77.1)	10,457 (76.6)
Gastrointestinal diseases	1,773 (3.3)	2,419 (4.6)	1,064 (7.8)	1,117 (8.2)
Psychiatric diseases	17,920 (33.8)	18,145 (34.2)	6,813 (49.9)	6,841 (50.1)
Neurological infections	143 (0.3)	262 (0.5)	95 (0.7)	96 (0.7)
**Employment**
Employed	21,743 (41.0)	21,584 (40.7)	4,488 (32.9)	4,481 (32.8)
Unemployed	2,057 (3.9)	2,379 (4.5)	606 (4.4)	652 (4.8)
Early retirement	7,611 (14.3)	7,705 (14.5)	2,346 (17.2)	2,387 (17.5)
State pensioner	21,678 (40.8)	21,421 (40.3)	6,216 (45.5)	6,136 (44.9)
**Income^a^**
Low	13,657 (25.7)	13,624 (25.7)	3,231 (23.7)	3,242 (23.7)
Intermediate	14,292 (26.9)	14,422 (27.2)	2,468 (18.1)	2,406 (17.6)
High	13,452 (25.3)	13,409 (25.3)	3,771 (27.6)	3,813 (27.9)
Very High	11,688 (22.0)	11,634 (21.9)	4,186 (30.7)	4,195 (30.7)
**Education^a^**
Basic education	25,402 (47.8)	25,306 (47.7)	6,799 (49.8)	6,765 (49.5)
Youth education high school or similar	19,885 (37.5)	19,832 (37.4)	4,819 (35.3)	4,825 (35.3)
Higher education	7,802 (14.7)	7,951 (15.0)	2,038 (14.9)	2,066 (15.1)
**B12 treatment during follow-up**
No	49,463 (93.2)	27,310 (51.4)
Yes	3,626 (6.8)	25,779 (48.6)

Numbers in brackets are percentages. ^a^Percentages do not add up to 100 due to rounding. Convert P-B12 from pmol/L to ng/L by multiplying with 1.355.

### Primary P-B12 treatment cohort

The IRs for the primary outcome, Alzheimer’s disease, were slightly different between the B12 level groups, but only within the first two years of follow-up (Table 2). This difference was mainly driven by a twofold increase in IRs for patients with low P-B12 who received B12 treatment during follow-up. The IRs in the follow-up strata of 2–<7 and 7–15 years decreased for patients with low P-B12, regardless of treatment or not during follow-up. The adjusted HRs were centered around 1 for patients not treated during follow-up, except for the first two years of follow-up. We observed a decreasing trend over follow-up time for patients who received B12 treatment. Estimates were similar for the secondary outcomes, all-cause dementia and vascular dementia (Supplementary Table 3). Overall, sex-stratified analyses showed very little difference by sex on the risk estimates (data not shown).

**Table 2:** IRs and adjusted HRs (95% CI) for the risk of Alzheimer’s disease in the primary P-B12 cohort, according to subsequent B12 treatment and follow-up time

	Follow-up (y)	P-B12 measurement P-B12 levels (pmol/L)		P-B12 measurement + B12-treatment during follow-up P-B12 levels (pmol/L)		P-B12 measurement + no B12-treatment during follow-up P-B12 levels (pmol/L)	
		200–600	<200	200–600	<200	200–600	<200
		n = 53,089	*n* = 53,089	*n* = 3,626	*n* = 25,779	*n* = 49,463	*n* = 27,310
No. with AD		1,058	1,176	112	658	946	518
IR/1000 PY		3.60 (3.38–3.82)	4.07 (3.83–4.30)	4.25 (3.46–5.03)	4.31 (3.98–4.64)	3.54 (3.31–3.76)	3.79 (3.46–4.11)
Adj. HR		ref.	1.12 (1.03–1.22)	ref.	1.15 (0.94–1.41)	ref.	1.23 (1.11–1.37)
	**0**–< **2**	***n* = 53,089**	***n* = 53,089**	***n* = 3,626**	***n* = 25,779**	***n* = 49,463**	***n* = 27,310**
No. with AD		518	647	20	320	498	327
IR/1000 PY		5.26 (4.81–5.71)	6.58 (6.07–7.09)	2.83 (1.59–4.07)	6.55 (5.83–7.27)	5.45 (4.97–5.93)	6.61 (5.90–7.33)
Adj. HR		ref.	1.22 (1.09–1.37)	ref.	2.53 (1.61–3.99)	ref.	1.36 (1.19-1.57)
	**2**–< **7**	***n* = 44,585**	***n* = 44,320**	***n* = 3,398**	***n* = 22,490**	***n* = 41,187**	***n* = 21,830**
No. with AD		404	421	59	262	345	159
IR/1000 PY		2.69 (2.43–2.96)	2.86 (2.59–3.13)	4.36 (3.25–5.47)	3.36 (2.95–3.77)	2.53 (2.26–2.80)	2.30 (1.94–2.65)
Adj. HR		ref.	1.07 (0.94–1.23)	ref.	0.91 (0.69–1.22)	ref.	1.11 (0.92–1.34)
	**7**–**15**	***n* = 16,124**	***n* = 15,687**	***n* = 1,819**	***n* = 8,766**	***n* = 14,305**	***n* = 6,921**
No. with AD		136	108	33	76	103	32
IR/1000 PY		3.00 (2.49–3.50)	2.47 (2.00–2.93)	5.71 (3.76–7.65)	2.96 (2.29–3.62)	2.60 (2.10–3.10)	1.77 (1.16–2.39)
Adj. HR		ref.	0.87 (0.68–1.12)	ref.	0.68 (0.45–1.02)	ref.	0.92 (0.62–1.38)

Convert P-B12 from pmol/L to ng/L by multiplying with 1.355. IR, incidence rate; HR, hazard ratio; PY, person-years; AD, Alzheimer’s disease.

### Secondary B12 treatment cohort

The IRs for the primary outcome, Alzheimer’s disease in the secondary B12 treatment cohort were similar among low and normal B12 level groups for all follow-up strata, and all HRs centered around 1 (Table 3). Results for the secondary outcomes, all-cause dementia and vascular dementia were similar to the primary outcome (Supplementary Table 4). Sex-stratified analyses showed very little difference by sex on the risk estimates (data not shown).

**Table 3:** IRs and adjusted HRs (95% CI) for the risk of Alzheimer’s disease in the secondary B12 treatment cohort, according to follow-up time and with and without propensity-score matching

	Follow-up (y)	B12-treatment		B12-treatment	
	Overall	Propensity-score matched P-B12 levels (pmol/L)		Not propensity-score matched P-B12 levels (pmol/L)	
		200–600	<200	200–600	<200
		(*n* = 13,656)	(*n* = 13,656)	(*n* = 13,717)	(*n* = 33,631)
No. with AD		332	324	333	876
IR/1000 PY		4.87 (4.34–5.39)	4.51 (4.02–5.00)	4.84 (4.32–5.36)	5.48 (5.12–5.84)
Adj. HR		ref.	1.03 (0.88–1.20)	ref.	1.00 (0.88–1.14)
	**0**–< **2**	(***n*** = **13,656)**	(***n*** = **13,656)**	(***n*** = **13,717)**	(***n*** = **33,631)**
No. with AD		172	163	172	519
IR/1000 PY		6.94 (5.91–7.98)	6.46 (5.47–7.46)	6.91 (5.88–7.94)	8.43 (7.70-9.15)
Adj. HR		ref.	1.01 (0.82–1.25)	ref.	1.07 (0.90-1.27)
	**2**–< **7**	(***n*** = **10,928)**	(***n*** = **11,249)**	(***n*** = **10,979)**	(***n*** = **26,963)**
No. with AD		126	127	126	290
IR/1000 PY		3.64 (3.00–4.27)	3.49 (2.88–4.09)	3.61 (2.98–4.24)	3.61 (3.20–4.03)
Adj. HR		ref.	1.03 (0.81–1.32)	ref.	0.90 (0.73–1.12)
	**7**–**15**	(***n*** = **3,215)**	(***n*** = **3,624)**	(***n*** = **3,250)**	(***n*** = **7,305)**
No. with AD		34	34	35	67
IR/1000 PY		3.87 (2.57–5.17)	3.33 (2.21–4.45)	3.89 (2.60–5.18)	3.72 (2.83–4.61)
Adj. HR		ref.	1.07 (0.67–1.73)	ref.	0.99 (0.65–1.49)

Convert P-B12 from pmol/L to ng/L by multiplying with 1.355. IR, incidence rate; HR, hazard ratio; PY, person-years; AD, Alzheimer’s disease. The following variables were included in the propensity score model: gender, year of P-B12 measurement, age, and the following diseases: cardiovascular diseases, gastrointestinal diseases, neurological infections, Parkinson’s disease, epilepsy, and psychiatric diseases. The same variables were adjusted for in Cox regression model in the not-propensity score-matched population.

### Subanalyses

We found no effect of any of the three specific B12 drugs on the primary outcome, Alzheimer’s disease risk according to pre-treatment P-B12 levels, except for injection therapy with 1 mg cyano-B12 in oil suspension, which showed a tendency toward lower IRs and HRs with longer follow-up. However, risk estimates were imprecise with wide CIs (Table 4).

**Table 4:** IRs and adjusted HRs with (95% CI) for the risk of Alzheimer’s disease in the secondary B12 treatment cohort with propensity-score matching according to type of B12 drug and follow-up time

	Follow-up (y)			Injection cyano-B12 1 mg, oil		Injection hydroxo-B12 1 mg,	
	Overall	Oral cyano-B12 1 mg		suspension		aqueous solution	
		P-B12 levels (pmol/L)		P-B12 levels (pmol/L)		P-B12 levels (pmol/L)	
		200–600	<200	200–600	<200	200–600	<200
		(*n* = 4,291)	(*n* = 4,291)	(*n* = 6,518)	(*n* = 6,518)	(*n* = 2,588)	(*n* = 2,588)
No. with AD		76	76	154	141	97	102
IR/1000 PY		3.71 (2.88–4.55)	3.68 (2.85–4.51)	4.85 (4.09–5.62)	4.15 (3.47–4.84)	6.54 (5.24–7.84)	6.37 (5.13–7.60)
Adj. HR		ref.	1.02 (0.75–1.41)	ref.	0.96 (0.76–1.21)	ref.	0.98 (0.74–1.29)
	**0**–< **2**	(***n*** = **4,291)**	(***n*** = **4,291)**	(***n*** = **6,518)**	(***n*** = **6,518)**	(***n*** = **2,588)**	(***n*** = **2,588)**
No. with AD		44	47	79	85	47	51
IR/1000 PY		5.60 (3.95–7.26)	5.93 (4.24–7.63)	6.59 (5.13–8.04)	6.96 (5.48–8.44)	10.46 (7.47–13.45)	11.05 (8.02–14.08)
Adj. HR		ref.	1.09 (0.72–1.65)	ref.	1.13 (0.83–1.54)	ref.	1.08 (0.72–1.60)
	**2**–< **7**	(***n*** = **3,361)**	(***n*** = **3,398)**	(***n*** = **5,427)**	(***n*** = **5,577)**	(***n*** = **1,951)**	(***n*** = **2,051)**
No. with AD		**≤**30^*^	**≤**30^*^	60	48	37	34
IR/1000 PY		2.67 (1.66–3.68)	2.56 (1.58–3.54)	3.62 (2.71–4.54)	2.71 (1.95–3.48)	5.01 (3.40–6.63)	4.27 (2.83–5.70)
Adj. HR		ref.	0.97 (0.57–1.67)	ref.	0.83 (0.56–1.21)	ref.	0.83 (0.52–1.32)
	**7**–**15**	(***n*** = **929)**	(***n*** = **933)**	(***n*** = **1,319)**	(***n*** = **1,658)**	(***n*** = **924)**	(***n*** = **1,035)**
No. with AD		**≤**5^*^	**≤**5^*^	15	8	13	17
IR/1000 PY		2.00 (0.25–3.76)	1.17 (0.00–2.50)	4.72 (2.33–7.11)	1.97 (0.60–3.34)	4.40 (2.01–6.79)	4.96 (2.60–7.31)
Adj. HR		ref.	0.68 (0.16–2.88)	ref.	0.59 (0.25–1.39)	ref.	1.07 (0.52–2.21)

^*^According to Danish Data legislation, observations≤5 are not allowed to display directly or indirectly. Convert P-B12 from pmol/L to ng/L by multiplying with 1.355. IR, incidence rate; HR, hazard ratio; PY, person-years; AD, Alzheimer’s disease.

Higher P-MMA levels were associated with a higher IRs of Alzheimer’s disease, mainly within the first two years of follow-up. After adjusting for potential confounders, the HRs centered around 1 and revealed no dose-response association between P-MMA levels and risk of Alzheimer’s disease (Table 5).

**Table 5:** IRs and adjusted HRs with corresponding 95% CIs for the risk of Alzheimer’s disease in the primary P-B12 cohort and in the secondary B12 treatment cohort, according to P-MMA levels and follow-up time

		Primary P-B12 cohort P-MMA levels (μmol/L)			Secondary B12 treatment cohort P-MMA levels (μmol/L)		
	Follow-up (y)	≤0.28	0.29–0.45	≥0.46	≤0.28	0.29–0.45	≥0.46
	Overall	(*n* = 39,995)	(*n* = 8,672)	(*n* = 2,963)	(*n* = 9,105)	(*n* = 6,109)	(*n* = 2,942)
No. with AD		673	252	86	149	188	96
IR/1000 PY		2.69 (2.49–2.89)	5.47 (4.80–6.15)	5.75 (4.54–6.97)	3.27 (2.75–3.80)	6.41 (5.50–7.33)	6.78 (5.42–8.13)
Adj. HR		ref.	1.17 (1.01–1.35)	0.98 (0.78–1.23)	ref.	1.25 (1.01–1.55)	1.25 (0.97–1.62)
	**0**–< **2**	**(*n*** = **39,995)**	**(*n*** = **8,672)**	**(*n*** = **2,963)**	**(*n*** = **9,105)**	**(*n*** = **6,109)**	**(*n*** = **2,942)**
No. with AD		343	150	50	80	103	62
IR/1000 PY		4.56 (4.07–5.04)	9.48 (7.96–11.00)	9.37 (6.77–11.97)	4.69 (3.66–5.72)	9.20 (7.42–10.98)	11.71 (8.80–14.63)
Adj. HR		ref.	1.18 (0.97–1.43)	0.91 (0.68–1.23)	ref.	1.23 (0.91–1.65)	1.44 (1.03–2.02)
	**2**–< **7**	**(*n*** = **34,507)**	**(*n*** = **6,962)**	**(*n*** = **2,321)**	**(*n*** = **7,597)**	**(*n*** = **4,868)**	**(*n*** = **2,288)**
No. with AD		218	75	28	53	71	26
IR/1000 PY		1.76 (1.53–2.00)	3.24 (2.51–3.97)	3.74 (2.35–5.12)	2.31 (1.69–2.93)	4.77 (3.66–5.89)	3.67 (2.26–5.08)
Adj. HR		ref.	1.12 (0.86–1.46)	1.07 (0.72–1.60)	ref.	1.33 (0.93–1.90)	0.99 (0.62–1.59)
	**7**–**15**	**(*n*** = **15,550)**	**(*n*** = **2,576)**	**(*n*** = **817)**	**(*n*** = **2,172)**	**(*n*** = **1,351)**	**(*n*** = **695)**
No. with AD		112	27	8	16	14	8
IR/1000 PY		2.19 (1.78–2.59)	3.82 (2.38–5.26)	3.75 (1.15–6.35)	2.90 (1.48–4.32)	4.30 (2.05–6.56)	4.47 (1.37–7.56)
Adj. HR		ref.	1.18 (0.77–1.80)	1.03 (0.50–2.12)	ref.	1.10 (0.53–2.30)	1.06 (0.45–2.51)

Convert P-B12 from pmol/L to ng/L by multiplying with 1.355. IR, incidence rate; HR, hazard ratio; MMA, methylmalonic acid; PY, person-years; AD, Alzheimer’s disease.

### Sensitivity analyses

Without propensity score matching, results were similar compared to the main analysis, except for higher IRs for patients with P-B12 values <200 pmol/L (Table 4). The same was found in the drug-specific analyses (data not shown) and for the secondary outcomes, all-cause dementia and vascular dementia (Supplementary Table 4).

When adding data on non-reimbursed B12 drug prescriptions to the primary cohort, 79 patients were excluded due to B12 treatment before P-B12 measurement; 57 among those with normal P-B12 and 22 among those with low P-B12. A total of 2,312 patients were re-classified as B12 treated during follow-up; 354 among those with normal P-B12 and 1,958 among those with low P-B12. Estimates of IRs and HRs were very similar to the results of the primary analysis, with only reimbursed B12 prescriptions (data not shown).

## DISCUSSION

In a large population-based cohort study in Northern and Central Denmark, we found that low P-B12 levels and high P-MMA levels were not associated with risk of Alzheimer’s disease, all-cause dementia, or vascular dementia. Moreover, high-dose B12 treatment did not alter these associations regardless of pre-treatment P-B12 or P-MMA levels, and regardless of the specific B12 treatment.

We found higher IRs of Alzheimer’s disease among patients with low P-B12 in the primary P-B12 cohort during short-term follow-up and particularly among those treated with high-dose B12. This finding could be due to confounding by indication for initiating B12 treatment, since higher risk was associated with high-dose treatment. We also observed lower IRs with longer follow-up among B12-treated patients with a low P-B12 level, a finding that suggests a possible compensatory deficit for the higher rates found within the first two years. The results from the secondary cohort support this view, showing no effect of pre-treatment P-B12 or P-MMA levels on risk of Alzheimer’s disease during both long and short-term follow-up. Findings were similar for all-cause dementia and vascular dementia. Taken together, these results suggest that B12 deficiency is not an important risk factor for Alzheimer’s disease or other dementias and that B12 treatment is ineffective for prevention, even in B12 deficient patients.

A recent meta-analysis, primarily involving people without dementia, did not find an effect of B vitamin treatment in any cognitive domain [6] and another meta-analysis concluded that B12 treatment does not affect cognitive performance [7] or prevent cognitive decline [9]. Smith and Refsum questioned these conclusions by arguing that most studies did not effectively identify the population at risk, namely those who are B vitamin deficient [10]. However, we found no association between dementia and B12 treatment in the at-risk population with low P-B12 or high P-MMA, nor could we show any specific effect of three different high-dose B12 drugs. Our results suggest that screening for and treating B12 deficiency have no effect on the risk of Alzheimer’s disease. However, we did not include direct measures of cognitive function.

Earlier clinical guidelines published by the UK National Institute for Health and Care Excellence recommended routine screening for B12 deficiency among patients suspected of dementia [27]. This recommendation was not included in the recently updated UK guideline [28]. However, the American Academy of Neurology and European guidelines recommend the routine assessment of B12 levels [29, 30]. Our findings support the recent UK clinical guidelines [28] and suggest that European and US guidelines should follow suit.

Our study has limitations. We studied patients who had a P-B12 measurement without knowing the clinical indication for the test. This patient group might have different disease risk profiles compared to the general population and also according to P-B12 levels. In this context, potential bias due to patient selection relates closely to the confounding effect of indication, a concern in observational studies. Neurological or psychological symptoms could be an indication to measure P-B12 levels and possibly also to initiate B12 treatment, as recommended in earlier guidelines [27, 29, 30]. However, previous studies have suggested that P-B12 measurements are requested without clear indication for the majority of patients [31, 32]. Although, we were unable to assess the indication for measuring P-B12 levels, we accounted for differences in comorbidity by propensity score matching and we adjusted for socioeconomic factors. We cannot preclude that specific patient sub-groups may have a particular indication for measuring and treating B12 deficiency, and that a small sub-group may benefit from B12 treatment in the prevention of dementia. Moreover, we were unable to include data on homocysteine and holo-transcobalamin as biomarkers for B12 deficiency.

A particular strength of our study is the good comparability of P-B12 measurements [33]. Hence, misclassification of P-B12 levels due to changes in analytical methods is unlikely. Further, we included two different data sources to identify persons treated with high-dose B12 drugs. Although high-dose B12 is available over-the-counter, we consider it likely that patients with P-B12 measurements were prescribed B12 drugs if B12 deficiency was diagnosed. Moreover, the sensitivity analysis on non-reimbursed B12 prescriptions showed robust results. We observed a small difference between patients with low and normal P-B12 in the proportion of deaths during follow-up in the secondary B12-treatment cohort. This could introduce survivor bias. However, we consider the risk of selection bias due to loss to follow-up minimal since all Danish citizens are registered in the Danish Civil Registration System, including date of death or emigration, allowing for censoring in the Cox regression analysis [12]. Another strength is the high positive predictive value of dementia (86%) and Alzheimer’s disease (81%) [34] in the DNPR, although less so for other dementia subtypes. The robustness of the results in subanalyses and sensitivity analyses also strengthens our findings. Although hydroxo-B12 is more readily converted to biologically important forms of B12 (methylcobalamin and adenosylcobalamin) than cyano-B12 [1], our results did not show any specific benefit of any formulation.

In conclusion, these robust results in a large patient population suggest that B12 deficiency does not contribute to the risk of Alzheimer’s disease or other dementia types, and that high-dose B12 treatment does not prevent these diseases regardless of P-B12 or P-MMA levels. Our findings from hospital inpatients, hospital outpatients, and general practitioner patients are highly representative of all Danish patients. These findings have high external validity at least for high-income countries. They have public health implications for the assessment of dementia and imply that guidelines suggesting routine screening for B12 deficiency in patients with suspected dementia should be reconsidered.

## Supplementary Material

Supplementary Material
