# Supplementary material for: Plasma Vitamin B12 Levels, High-Dose Vitamin B12 Treatment, and Risk of Dementia
Source: J Alzheimers Dis. 2021 Feb 16;79(4):1601–12. doi: 10.3233/JAD-201096 (PMC7990402; doi:10.3233/JAD-201096)
Supplement: Supplementary Material [file jad-79-jad201096-s001.pdf]

Supplementary Material

Plasma Vitamin B12 Levels, High-Dose Vitamin B12 Treatment, and Risk of Dementia

Supplementary Table 1. Overview of registers and databases, diagnosis codes (ICD-8 and ICD-10), drug codes (ATC) and laboratory codes (NPU and local codes) used in this study.

Registry	Codes	
**Danish National Registry of Patients AND Danish Psychiatric Central Research Register**	ICD-8 codes for dementia	Alzheimer's disease: 290.10, 290.09<br>Vascular dementia: 293.09, 293.19<br>Other dementia: 094.19 and 292.09, 290.11, 290.18, 290.19, 292.09<br>MCI/amnestic syndromes: 291.19
	ICD-10 codes for dementia	Alzheimer's disease: F00, G30<br>Vascular dementia: F01<br>Other dementia: B220.A, F02, F03, F05.1, F10.73, F.11.73, F12.73, F.13.73, F14.73, F.15.73, F16.73, F.17.73, F18.73, F.19.73 G23.1, G31.0, G31.1, G31.8B, G31.8E, G31.85<br>MCI/amnestic syndromes: F04, F04.9, F05.1, F06.7, F10.6, F18.6, F19.6
**Danish National Registry of Patients (for propensity scores)**	ICD-8 codes for cardiovascular diseases	Hypertension: 400-404; Ischemic heart disease: 410, 411, 413; Endocarditis: 421; Non-rheumatic valve diseases 394-395; Atrial fibrillation/flutter: 427.93, 427.94; Other arrhythmias: 427.20, 427.21, 427.22, 427.23, 427.97, 427.91; Heart failure: 427.09, 427.10, 427.11, 427.19, 428.99, 782.49; Hypercholesterolemia: 272.00; Stroke: 430, 431, 433-435
	ICD-10 codes cardiovascular diseases	Rheumatic valve diseases: I05-09; Hypertension: I10-12, I13; Ischemic heart disease: I20-25; Endocarditis: I33; Non-rheumatic valve diseases I34-37, I39; Cardiomyopathy: I42; Atrial fibrillation/flutter: I48; Other arrhythmias: I44-45, I47, I49; Congestive heart failure: I50; Hypercholesterolemia: E78.0; Stroke: I60-61, I63-64, G45.9
	ICD-8 codes for gastrointestinal diseases	Inflammatory bowel disease: 563; Chronic liver disease: 070, 571; Chronic cholecystitis and pancreatitis: 575, 577
	ICD-10 codes for gastrointestinal diseases	Inflammatory bowel disease: K50-51, K52.8; Alcoholic liver disease: K70; Chronic hepatitis: K71.3-71.7, K73, K75.4; Liver insufficiency and fibrosis: K72.1-72.9, K74; Chronic cholecystitis and pancreatitis: K80.1, K81.1, K83.0F, K86.0, K86.1
	ICD-8 codes for psychiatric diseases	Psychoses and neuroses: 291-299; 300; Personality disorders; 301; Mental retardation: 310-315
	ICD-10 codes psychiatric diseases	Organic types of psychiatric diseases other than dementia: F05-09; Schizophrenia, affective disorders, anxiety: F20-42; Pregnancy-related psychiatric disorders: F53; Personality disorders: F60-69; Mental retardation F70-79; Developmental disorders: F80-89; Behavioral and emotional disorders: F90-98
	ICD-8 codes for neurological diseases	Parkinson's disease: 342, 066. Epilepsy: 345, 331.29. Neurological infections: 036, 040-046, 071, 094, 320-324
	ICD-10 codes neurological diseases	Parkinson's disease: G20, G21.3-G21.9, G22. Epilepsy: G40, G41. Neurological infections: A02.2C, A17.0, A20.3, A32.1, A39.0, A52.1-3, A54.8D, A81-89, B00.3, B01.0, B02.1-2, B05.0-1, B22.0, B26.1-2, B37.5, B38.4, B45.1, G00-G07

Registry	Codes	
**LABKA Database**	NPU/local Danish analysis codes for P-B12 measurement	NPU01700, AAA00281, AAA00304, 1700, 110212, 111212, 1316710, 131671, 1416710, 1416711, 1516710, 1610117, 1711700, 1811700
**Aarhus University Prescription Database**	ATC codes for B12 therapy	B03BA01: Oral cyanocobalamin 1 mg<br>B03BA02: Injection cyanocobalamin 1 mg, oil suspension,<br>B03BA03: Injection hydroxocobalamin 1 mg, aqueous solution
	ATC codes for cardiovascular drugs	Anti-arrhythmias: C01; Anti-hypertensives: C02-05, C07-09; Other; C10
	ATC codes for gastrointestinal drugs	Anti-inflammatory gastrointestinal drugs: A07E;
	ATC codes for neuropsychiatric drugs	Anti-epilepsy: N03; Anti-Parkinson: N04; Psycholeptica: N05A-B, N05CD, N05CF; Antidepressant: N06A, N06CA
**Danish National Prescription Registry**	ATC codes for B12 therapy	B03BA01: Oral cyanocobalamin 1 mg<br>B03BA02: Inj. cyanocobalamin 1 mg, oil suspension,<br>B03BA03: Inj. hydroxocobalamin 1 mg, aqueous solution

Supplementary Table 2. Basic characteristics of the not-propensity score-matched secondary B12 treatment cohort, Denmark, 2000-2013.

Not-propensity score-matched secondary B12 treatment cohort

	**P-B12 levels (pmol/L)**	
	**200-600**	**<200**
**Total**	13,717 (100.0)	33,631 (100.0)
**Sex, female**	8,342 (60.8)	19,178 (57.0)
**Age**[a]		
40-60 y		11,090 (33.0)
61-80 y	6,210 (45.3)	16,648 (49.5)
≥81 y	2,094 (15.3)	5,893 (17.5)
**Year of B12 treatment**		
2000-2004	2,661 (19.4)	4,564 (13.6)
2005-2009	5,736 (41.8)	13,323 (39.6)
2010-2013	5,320 (38.8)	15,744 (46.8)
**Died during follow-up**		
No	10,305 (75.1)	26,968 (80.2)
Yes	3,412 (24.9)	6,663 (19.8)
**Previous diagnoses**		
Cardiovascular diseases	10,585 (77.2)	24,780 (73.7)
Gastrointestinal diseases	1,074 (7.8)	2,251 (6.7)
Psychiatric diseases	6,865 (50.0)	14,189 (42.2)
Neurological infections	95 (0.7)	182 (0.5)
**Employment**[a]		
Employed	4,501 (32.8)	10,260 (30.5)
Unemployed	606 (4.4)	1,335 (4.0)
Early retirement	2,346 (17.2)	4,898 (14.6)
State pensioner	6,246 (45.5)	17,138 (51.0)
**Income**		
Low	3,256 (23.7)	7,955 (23.7)
Intermediate	2,477 (18.1)	6,693 (19.9)
High	3,785 (27.6)	9,319 (27.7)
Very High	4,199 (30.6)	9,664 (28.7)
**Education**[a]		
Basic education	6,828 (49.8)	17,333 (51.5)
Youth education high school or similar	4,839 (35.3)	11,889 (35.4)
Higher education	2,050 (14.9)	4,409 (13.1)

Numbers in brackets are percentages. [a]Percentages do not add up to 100 due to rounding. Convert P-B12 from pmol/L to ng/L by multiplying with 1.355.

Supplementary Table 3. IRs and adjusted HRs (95% CI) for the risk of all-cause dementia and vascular dementia in the primary P-B12 cohort, according to subsequent B12 treatment and follow-up time.

	Follow-up (y)	P-B12 measurement P-B12 levels (pmol/L)		P-B12 measurement + B12-treatment during follow-up P-B12 levels (pmol/L)		P-B12 measurement + no B12-treatment during follow-up P-B12 levels (pmol/L)	
		**200-600**	**<200**	**200-600**	**<200**	**200-600**	**<200**
	**Overall**	**n=53,089**	**n=53,089**	**n=3,636**	**n=25,779**	**n=49,463**	**n=27,310**
**No. with all-cause dementia**		2,354	2,786	237	1,517	2,117	1,269
**IR/1000 PY**		8.01 (7.69-8.34)	9.63 (9.27-9.99)	8.98 (7.84-10.13)	9.95 (9.45-10.45)	7.92 (7.58-8.25)	9.28 (8.77-9.79)
**Adj. HR**		ref.	1.20 (1.13-1.26)	ref.	1.33 (1.16-1.53)	ref.	1.35 (1.26-1.45)
	**0-<2**	**n=53,089**	**n=53,089**	**n=3,636**	**n=25,779**	**n=49,463**	**n=27,310**
**No. with all-cause dementia**		1,117	1,429	52	658	1,065	771
**IR/1000 PY**		11.35 (10.68-12.01)	14.54 (13.78-15.29)	7.35 (5.35-9.35)	13.47 (12.44-14.49)	11.66 (10.96-12.36)	15.59 (14.49-16.69)
**Adj. HR**		ref.	1.25 (1.16-1.36)	ref.	2.12 (1.60-2.82)	ref.	1.50 (1.37-1.65)
	**2-<7**	**n=44,585**	**n=44,320**	**n=3,398**	**n=22,490**	**n=41,187**	**n=21,830**
**No. with all-cause dementia**		940	1,052	125	645	815	407
**IR/1000 PY**		6.27 (5.87-6.67)	7.15 (6.71-7.58)	9.24 (7.62-10.86)	8.27 (7.63-8.91)	5.97 (5.56-6.38)	5.88 (5.31-6.45)
**Adj. HR**		ref.	1.15 (1.05-1.25)	ref.	1.12 (0.92-1.36)	ref.	1.19 (1.06-1.34)
	**7-15**	**n=16,124**	**n=15,687**	**n=1,819**	**n=8,766**	**n=14,305**	**n=6,921**
**No. with all-cause dementia**		297	305	60	214	237	91
**IR/1000 PY**		6.54 (5.80-7.29)	6.97 (6.19-7.76)	10.38 (7.75-13.00)	8.33 (7.22-9.45)	5.98 (5.22-6.75)	5.04 (4.00-6.07)
**Adj. HR**		ref.	1.12 (0.96-1.32)	ref.	1.05 (0.79-1.40)	ref.	1.12 (0.88-1.43)
	**Overall**	**n=53,089**	**n=53,089**	**n=3,636**	**n=25,779**	**n=49,463**	**n=27,310**
**No. with vascular dementia**		357	407	39	227	318	180
**IR/1000 PY**		1.22 (1.09-1.34)	1.4 (1.27-1.54)	1.48 (1.01-1.94)	1.49 (1.29-1.68)	1.19 (1.06-1.32)	1.32 (1.12-1.51)
**Adj. HR**		ref.	1.15 (1.00-1.32)	ref.	1.29 (0.92-1.82)	ref.	1.27 (1.05-1.52)
	**0-<2**	**n=53,089**	**n=53,089**	**n=3,636**	**n=25,779**	**n=49,463**	**n=27,310**
**No. with vascular dementia**		171	196	11	91	160	105
**IR/1000 PY**		1.74 (1.48-2.00)	1.99 (1.71-2.27)	1.56 (0.64-2.47	1.86 (1.48-2.24)	1.75 (1.48-2.02)	2.12 (1.72-2.53)
**Adj. HR**		ref.	1.12 (0.91-1.37)	ref.	1.5 (0.80-2.83	ref.	1.34 (1.05-1.71)
	**2-<7**	**n=44,585**	**n=44,320**	**n=3,398**	**n=22,490**	**n=41,187**	**n=21,830**
**No. with vascular dementia**		139	156	20	95	119	61
**IR/1000 PY**		0.93 (0.77-1.08)	1.06 (0.89-1.23)	1.48 (0.83-2.13)	1.22 (0.97-1.46)	0.87 (0.72-1.03)	0.88 (0.66-1.10)
**Adj. HR**		ref.	1.15 (0.91-1.44)	ref.	1.08 (0.66-1.75)	ref.	1.22 (0.90-1.67)
	**7-15**	**n=16,124**	**n=15,687**	**n=1,819**	**n=8,766**	**n=14,305**	**n=6,921**
**No. with vascular dementia**		47	55	8	41	39	14
**IR/1000 PY**		1.04 (0.74-1.33)	1.26 (0.93-1.59)	1.38 (0.42-2.34)	1.60 (1.11-2.09)	0.98 (0.68-1.29)	0.78 (0.37-1.18)

Convert P-B12 from pmol/L to ng/L by multiplying with 1.355. IR, incidence rate; HR, hazard ratio; PY, person-years.

Supplementary Table 4. Incidence rates and adjusted hazard ratios (95% CI) for the risk of all-cause dementia and vascular dementia in the secondary B12 treatment cohort, according to follow-up time and with and without propensity-score matching.

		**B12-treatment Propensity-score matched P-B12 levels (pmol/L)**		**B12-treatment Not propensity-score matched P-B12 levels (pmol/L)**	
	**Follow-up (y) Overall**	**200-600 (n=13,656)**	**<200 (n=13,656)**	**200-600 (n=13,717)**	**<200 (n=33,631)**
**No. with all-cause dementia**		830	796	836	2,042
**IR/1000 PY**		12.17 (11.34-12.99)	11.08 (10.31-11.85)	12.16 (11.34-12.99)	12.77 (12.22-13.33)
**Adj. HR**		ref.	1.00 (0.91-1.10)	ref.	0.98 (0.91-1.07)
	**0-<2**	**(n=13,656)**	**(n=13 656)**	**(n=13,717)**	**(n=33,631)**
**No. with all-cause dementia**		419	388	420	1,132
**IR/1000 PY**		16.91 (15.29-18.53)	15.39 (13.86-16.92)	16.88 (15.26-18.49)	18.38 (17.31-19.45)
**Adj. HR**		ref.	0.98 (0.85-1.12)	ref.	1.01 (0.90-1.13)
	**2-<7**	**(n=10,928)**	**(n=11,249)**	**(n=10,979)**	**(n=26,963)**
**No. with all-cause dementia**		321	322	324	748
**IR/1000 PY**		9.26 (8.25-10.28)	8.84 (7.88-9.81)	9.29 (8.28-10.30)	9.32 (8.65-9.99)
**Adj. HR**		ref.	1.02 (0.88-1.19)	ref.	0.95 (0.83-1.08)
	**7-15**	**(n=3,215)**	**(n=3,624)**	**(n=3,250)**	**(n=7,305)**
**No. with all-cause dementia**		90	86	92	162
**IR/1000 PY**		10.24 (8.13-12.36)	8.43 (6.65-10.21)	10.24 (8.15-12.33)	8.99 (7.60-10.37)
**Adj. HR**		ref.	1.02 (0.76-1.37)	ref.	0.95 (0.73-1.23)
	**Follow-up interval Overall**	**P-B12 levels (pmol/L) 200-600 (n=13,656)**	**<200 (n=13,656)**	**P-B12 levels (pmol/L) 200-600 (n=13,717)**	**<200 (n=33,631)**
**No. with vascular dementia**		144	136	144	320
**IR/1000 PY**		2.11 (1.77-2.46)	1.89 (1.58-2.21)	2.09 (1.75-2.44)	2.00 (1.78-2.22)
**Adj. HR**		ref.	0.98 (0.78-1.24)	ref.	0.95 (0.78-1.16)
	**0-<2**	**(n=13,656)**	**(n=13,656)**	**(n=13,717)**	**(n=33,631)**
**No. with vascular dementia**		77	66	77	172
**IR/1000 PY**		3.11 (2.41-3.80)	2.62 (1.99-3.25)	3.09 (2.40-3.79)	2.79 (2.38-3.21)
**Adj. HR**		ref.	0.90 (0.65-1.25)	ref.	0.91 (0.69-1.19)
	**2-<7**	**(n=10,928)**	**(n=11,249)**	**(n=10,979)**	**(n=26,963)**
**No. with vascular dementia**		52	53	52	122
**IR/1000 PY**		1.50 (1.09-1.91)	1.46 (1.06-1.85)	1.49 (1.09-1.90)	1.52 (1.25-1.79)
**Adj. HR**		ref.	1.04 (0.71-1.53)	ref.	1.02 (0.73-1.41)
	**7-15**	**(n=3,215)**	**(n=3,624)**	**(n=3,250)**	**(n=7,305)**
**No. with vascular dementia**		15	17	15	26
**IR/1000 PY**		1.71 (0.84-2.57)	1.67 (0.87-2.46)	1.67 (0.82-2.51)	1.44 (0.89-2.00)

Convert P-B12 from pmol/L to ng/L by multiplying with 1.355. IR, incidence rate; HR, hazard ratio; PS, propensity score; PY, person-years.

The following variables were included in the propensity score model: gender, year of P-B12 measurement, age, and the following diseases: cardiovascular diseases, gastrointestinal diseases, neurological infections, Parkinson's disease, epilepsy, and psychiatric diseases. The same variables were adjusted for in Cox regression model in the not-propensity score-matched population.
